# Supplementary material for: Antimicrobial Resistance in Physiological and Potentially Pathogenic Bacteria Isolated in Southern Italian Bats
Source: Animals (Basel). 2023 Mar 7;13(6):966. doi: 10.3390/ani13060966 (PMC10044454; doi:10.3390/ani13060966)
Supplement: Supplementary file 1 [file animals-13-00966-s001.zip › animals-2236547-supplementary.pdf]

Supplementary Table S1: Gram negative strains for different samples

Bacterial Family	Bacterial species	Number of strains			
		R	C	O	**Total**
Aeromonadaceae	*Aeromonas hydrophila*			3	3
	Aeromonas spp			1	1
Alcaligenaceae	*Achromobacter denitrificans*		1		1
	Achromobacter insolitus		1	1	2
	Alcaligenes faecalis		1		1
	Alcaligenes xylosoxidans			1	1
Enterobacteriaceae Group	*Citrobacter amalonaticus*	3		2	5
	Citrobacter farmeri	1		1	2
	Citrobacter freundii	16		5	21
	Citrobacter sedlakii	2			2
	Citrobacter spp	31	3	12	46
	Citrobacter youngae	1			1
	Cronobacter muytjensis		1		1
	Cronobacter zurichensis			1	1
	Enterobacter aerogenes	2	1	9	12
	Enterobacter agglomerans	2			2
	Enterobacter asburiae			1	1
	Enterobacter cancerogenus	11	2	6	19
	Enterobacter cloacae	38	5	28	71
	Enterobacter gergoviae			1	1
	Enterobacter kobei	4	1	5	10
	Enterobacter ludwigii	7	1	4	12
	Enterobacter spp	6	1	4	11
	Escherichia coli	6	1	6	13
	Hafnia alvei	19	4	24	47
	Klebsiella oxytoca	3		10	13
	Klebsiella pneumoniae	2	1	2	5
	Kluyvera intermedia			2	2
	Leclercia adecarboxilata			1	1
	Moellerella wisconsensis			2	2
	Morganella morganii	2	1	13	16
	Pantoea agglomerans		1		1
	Proteus mirabilis	1		4	5
	Proteus vulgaris	1	1		2
	Providencia alcalifaciens			2	2
	Providencia rettgeri	8	2	7	17
	Providencia rustigianii	1	1	1	3
	Providencia spp			1	1
	Rahnella aquatilis			1	1
	Salmonella Manhattan[a]	1			1
	Serratia liquefaciens	1		3	4
	Serratia marcescens	2		19	21
	Serratia plymuthica	1			1
	Shighella flexneri		1		1
Moraxellaceae	*Acinetobacter* spp.		1		1
Pasteurellaceae	*Pasteurella aerogenes*	1			1

Pseudomonadaceae	*Pseudomonas aeruginosa*	2		11	13
	Pseudomonas aureofaciens			1	1
	Pseudomonas spp		1		1
	Pseudomonas stutzeri		2	2	4
	Not id	1	1	4	6
Total		178	36	199	413

[a]Salmonella isolate named by the respective serotype of *Salmonella enterica* ssp. *enterica*.

Supplementary Table S2: Gram negative strains for species of bat

		Number of isolates				
Bacterial Family	Bacterial species	*Miniopterus schreibersii*	*Myotis capaccinii*	*Myotis myotis*	*Rhinolophus hipposideros*	Total
Aeromonadaceae	*Aeromonas hydrophila*	1			2	3
	Aeromonas spp		1			1
Alcaligenaceae	*Achromobacter denitrificans*				1	1
	Achromobacter insolitus	1			1	2
	Alcaligenes faecalis	1				1
	Alcaligenes xylosoxidans				1	1
Enterobacteriaceae Group	*Citrobacter amalonaticus*	1		4		5
	Citrobacter farmeri	2				2
	Citrobacter freundii	8	2	3	8	21
	Citrobacter sedlakii			2		2
	Citrobacter spp	19		17	10	46
	Citrobacter youngae	1				1
	Cronobacter muytjensis			1		1
	Cronobacter zurichensis			1		1
	Enterobacter aerogenes	2		9	1	12
	Enterobacter agglomerans	2				2
	Enterobacter asburiae				1	1
	Enterobacter cancerogenus	9		5	5	19
	Enterobacter cloacae	42		20	9	71
	Enterobacter gergoviae				1	1
	Enterobacter kobei	6		2	2	10
	Enterobacter ludwigii	6		4	2	12
	Enterobacter spp	8		2	1	11
	Escherichia coli	4		2	7	13
	Hafnia alvei	21	2	6	18	47
	Klebsiella oxytoca	2	2	2	7	13
	Klebsiella pneumoniae	1		4		5
	Kluyvera intermedia	1		1		2
	Leclercia adecarboxilata			1		1
	Moellerella wisconsensis				2	2
	Morganella morganii	8	1	1	6	16
	Pantoea agglomerans	1				1
	Proteus mirabilis	2		1	2	5
	Proteus vulgaris	1			1	2

	Providencia alcalifaciens	1			1	2
	Providencia rettgeri		1	8	8	17
	Providencia rustigianii	3				3
	Providencia spp			1		1
	Rahnella aquatilis	1				1
	Salmonella enterica			1		1
	Serratia liquefaciens	1	1	1	1	4
	Serratia marcescens	7	4	3	7	21
	Serratia plymuthica				1	1
	Shighella flexneri				1	1
Moraxellaceae	*Acinetobacter* spp.	1				1
Pasteurellaceae	*Pasteurella aerogenes*				1	1
Pseudomonadaceae	*Pseudomonas aeruginosa*	3		10		13
	Pseudomonas aureofaciens	1				1
	Pseudomonas spp	1				1
	Pseudomonas stutzeri	2			2	4
	Not id.	4	1		1	6
	Total	175/91	15/8	112/47	111/43	413

Supplementary Table S3: Gram positive strains for different samples.

Bacterial Family	Bacterial species	Number of isolates		
		C	O	Total
Aerococcaceae	*Aerococcus viridans*	1	2	3
Bacillaceae	*Bacillus amiloliquefaciens*	3	1	4
	Bacillus atrophaeus	1		1
	Bacillus brevis	1	1	2
	Bacillus cereus	2	3	5
	Bacillus circulans		2	2
	Bacillus clausii	2		2
	Bacillus coagulans	1		1
	Bacillus fastidiosus	1		1
	Bacillus firmus	1		1
	Bacillus laterosporus	1		1
	Bacillus licheniformis	4	11	15
	Bacillus macerans	1		1
	Bacillus megaterium	11	3	14
	Bacillus pantothenticus	1		1
	Bacillus pasteurii	1		1
	Bacillus pumilis	3	2	5
	Bacillus simplex	2		2
	Bacillus sphaericus	2		2
	Bacillus spp	2		2
	Bacillus subtilis	6	2	8
Enterococcaceae	*Enterococcus casseliflavus*	2	1	3
	Enterococcus faecalis	9	19	28
Lactobacillaceae	*Alloicoccus otitis*		1	1
Paenibacillaceae	*Paenibacillus durus*		1	1
Staphylococcaceae	*Staphylococcus aureus*		1	1

	Staphylococcus capitis	2	1	3
	Staphylococcus cohnii subsp. 1	2	2	4
	Staphylococcus cohnii subsp. 2	5		5
	Staphylococcus epidermidis	9		9
	Staphylococcus gallinarum	1	1	2
	Staphylococcus haemolyticus	2	2	4
	Staphylococcus hominis	1		1
	Staphylococcus koosi		3	3
	Staphylococcus lentus	2	5	7
	Staphylococcus saprophyticus	2	1	3
	Staphylococcus sciuri	1	11	12
	Staphylococcus simulans	2		2
	Staphylococcus warneri	8	2	10
	Staphylococcus xylosus	5	2	7
	Not id.	1	1	2
Total		101	81	183

Supplementary Table S4: Gram positive strains for species of bat

		Number of isolates				
Bacterial Family	Bacterial species	*Miniopterus schreibersii*	*Myotis capaccinii*	*Myotis myotis*	*Rhinolophus hipposideros*	Total
Aerococcaceae	*Aerococcus viridans*	2	1			3
Bacillaceae	*Bacillus amyloliquefaciens*	2		2		4
	Bacillus atrophaeus			1		1
	Bacillus brevis	1			1	2
	Bacillus cereus	1	1	1	2	5
	Bacillus circulans	2				2
	Bacillus clausii	1			1	2
	Bacillus coagulans			1		1
	Bacillus fastidiosus	1				1
	Bacillus firmus	1				1
	Bacillus laterosporus	1				1
	Bacillus licheniformis	7		3	5	15
	Bacillus macerans			1		1
	Bacillus megaterium	12		1	1	14
	Bacillus pantothenticus	1				1
	Bacillus pasteurii	1			1	2
	Bacillus pumilis	2		2	1	5
	Bacillus simplex				2	2
	Bacillus sphaericus	2				2
	Bacillus spp	2				2
	Bacillus subtilis	4		3	1	8
Enterococcaceae	*Enterococcus casseliflavus*		1	2		3
	Enterococcus faecalis	14	2	7	5	28
Lactobacillaceae	*Alloicoccus otitis*				1	1
Paenibacillaceae	*Paenibacillus durus*	1				1
Staphylococcaceae	*Staphylococcus aureus*			1		1
	Staphylococcus capitis	1			2	3
	Staphylococcus cohnii subsp. 1	1		2	1	4

	Staphylococcus cohnii subsp. 2	1		4		5
	Staphylococcus epidermidis	4	1	1	3	9
	Staphylococcus gallinarum	1		1		2
	Staphylococcus haemolyticus	3			1	4
	Staphylococcus hominis		1			1
	Staphylococcus koosi	3				3
	Staphylococcus lentus	2		3	2	7
	Staphylococcus saprophyticus	2		1		3
	Staphylococcus sciuri	3		7	2	12
	Staphylococcus simulans	2				2
	Staphylococcus warneri	8	1		1	10
	Staphylococcus xylosus	5	1		1	7
	Not id.	1	1			2
	Total	95/91	10/8	44/47	34/43	183/189

Supplementary Table S5: List of antibiotics used in Gram negative antimicrobial susceptibility tests and relative inhibition halos diameters for resistant (R), intermediate (I) and sensitive (S) strains.

**Class**	**Antibiotics**	Code µg	Diameter (mm)		
			R <	I	S >
Aminoglycosides	gentamicin	CN 10	13	14-15	16
	streptomycin	S 10	11	12-14	15
	tobramycin	TOB 10	13	14-15	16
Carbapenems	imipenem	IMI 10	19	20-22	23
	meropenem	MRP 10	19	20-22	23
Cephalosporins	cefotaxime	CTX 30	22	23-25	26
	cefotaxime+clavulanic acid	CTL 40	22	23-25	26
	ceftazidime	CAZ 30	17	18-20	21
Chloramphenicol	chloramphenicol	C 30	12	13-17	18
Fluoroquinolones	ciprofloxacin	CIP 5	19	20-21	22
	enrofloxacin	ENR 5	15	16-20	21
Monobactams	aztreonam	ATM 30	17	18-20	21
Penicillins	amoxicillin	AML 30	13	14-17	18
	amoxicillin+clavulanic acid	AUG 30	13	14-17	18
	ampicillin	AMP 10	13	14-16	17
Polymyxins	colistin sulfate	CS 10	11	12-16	17
Quinolones	nalidixic acid	NA 30	13	14-18	19
Sulfonamides	co-trimoxazole	SXT 25	13	14-15	16
Tetracyclines	minocycline	MN 30	12	13-15	16
	tetracycline	TE 30	11	12-14	15

Supplementary Table S6: List of antibiotics used in Gram positive antimicrobial susceptibility tests and relative inhibition halos diameters for resistant (R), intermediate (I) and sensitive (S) strains.

**Class**	**Antibiotics**	Code µg	Diameter (mm)		
			R <	I	S >
Aminoglycosides	gentamicin	CN 10	12	13-14	15
	tobramycin	TOB 10	12	13-14	15
Carbapenems	imipenem	IMI 10	16	17-21	22

	meropenem	MRP 10	16	17-21	22
Cephalosporins	ceftazidime	CAZ 30	14	15-17	18
	cefovecin	CVN 10	14	15-20	21
	cefepime	FEP 30	14	15-17	18
	ceftaroline	CPT 30	23		23
Fluoroquinolones	enrofloxacin	ENR 5	17	18-20	21
Glycopeptides	vancomycin	VA 30	14	15-16	17
Lincosamides	lincomycin	MY 15	14	15-20	21
Macrolides	erythromycin	E 15	13	14-22	23
Penicillins	amoxicillin	AML 30	13	14-16	17
	amoxicillin+clavulanic acid	AUG 30	19		20
	ampicillin<br>*Staphylococcus* spp.	AMP 10	13<br>28	14-16	17<br>29
	ampicillin+sulbactam	AMS 20	11	12-14	15
	oxacillin<br>*Staphylococcus* spp. coagulase -	OX 1	10<br>18	11-12	13<br>19
	ticarcillin+clavulanic acid	TIM 85	22		23
Tetracyclines	minocycline	MN 30	14	15-18	19
	tetracycline	TE 30	11	12-14	15

Supplementary Table S7: MDR pattern in Gram-negative strains.

N. Classes	Antimicrobial Classes	Bacterial Genera and n. strains ()	Total strains
8	AMI; CEP; QUI; PEN; SUL; TET; PHE; POL	*Pseudomonas* (2)	2
	AMI; CAR; QUI; PEN; SUL; TET; PHE; POL	*Pseudomonas* (1)	1
	AMI; CEP; CAR; QUI; MON; PEN; SUL; POL	*Klebsiella* (1)	1
	AMI; CEP; CAR; QUI; MON; PEN; TET; POL	*Citrobacter* (1)	1
7	AMI; CEP; CAR; QUI; PEN; TET; POL	*Citrobacter* (3), *Enterobacter* (2)	5
	AMI; QUI; PEN; SUL; TET; PHE; POL	*Pseudomonas* (3)	3
	AMI; CEP; QUI; PEN; SUL; TET; POL	*Achromobacter* (1)	1
	AMI; CEP; CAR; QUI; MON; TET; POL	*Enterobacter* (1)	1
	AMI; CEP; MON; PEN; SUL; PHE; POL	*Enterobacter* (1)	1
	AMI; CEP; CAR; PEN; SUL; TET; POL	*Enterobacter* (1)	1
	AMI; CEP; QUI; PEN; SUL; TET; POL	*Pseudomonas* (1)	1
	AMI; CEP; CAR; MON; PEN; TET; POL	*Citrobacter* (1)	1
6	AMI; CEP; CAR; PEN; TET; POL	*Citrobacter* (3), *Enterobacter* (6), *Proteus* (1), *Serratia* (2)	12
	AMI; CEP; CAR; QUI; MON; POL	*Enterobacter* (2), *Hafnia* (2), *Providencia* (2), *Serratia* (2)	8
	AMI; CEP; MON; PEN; PHE; POL	*Achromobacter* (1)	1
	AMI; QUI; PEN; TET; PHE; POL	*Pseudomonas* (1)	1
	CEP; CAR; MON; PEN; PHE; POL	*Pseudomonas* (1)	1
5	AMI; CAR; PEN; TET; POL	*Citrobacter* (1), *Enterobacter* (7), *Morganella* (1)	9
	AMI; CEP; PEN; TET; POL	*Enterobacter* (3), *Proteus* (1)	4
	AMI; CEP; QUI; MON; POL	*Enterobacter* (1), *Providencia* (1), *Serratia* (2)	4
	AMI; QUI; PEN; TET; POL	*Citrobacter* (1), *Pseudomonas* (3)	4
	AMI; QUI; MON; PEN; POL	*Kluyvera* (1)	1
	AMI; CAR; QUI; PEN; POL	*Moellerella* (1)	1

	AMI; CEP; CAR; PEN; POL	*Enterobacter* (1)	1
4	AMI; PEN; TET; POL	*Citrobacter* (1), *Enterobacter* (10), *Hafnia* (1), *Providencia* (1), *Pseudomonas* (1), *Serratia* (3)	17
	AMI; CEP; PEN; POL	*Alcaligenes* (2), *Citrobacter* (1), *Enterobacter* (1)	4
	AMI; CAR; PEN; POL	*Citrobacter* (2)	2
	AMI; MON; PEN; POL	*Citrobacter* (1)	1
	QUI; MON; PEN; POL	*Enterobacter* (1)	1
	CEP; MON; PEN; POL	*Enterobacter* (1)	1
	CEP; QUI; MON; POL	*Klebsiella* (1)	1
	CAR; PEN; TET; POL	*Morganella* (1)	1
3	AMI; PEN; POL	*Citrobacter* (5), *Enterobacter* (31), *Escherichia* (1), *Hafnia* (2), *Klebsiella* (2), *Morganella* (2), *NI* (1), *Proteus* (1), *Pseudomonas* (2), *Serratia* (3)	50
	PEN; TET; POL	*Enterobacter* (3), *Klebsiella* (2), *Moellerella* (1), *Morganella* (4), *Proteus* (1), *Providencia* (10), *Serratia* (3)	24
	QUI; MON; POL	*Enterobacter* (1)	1
	AMI; TET; POL	*Serratia* (1)	1

Legenda: AMI= Aminoglycosides; CEP= cephalosporin; CAR= carbapenemes; QUI= quinolones; MON= monobactams; PEN= penicillins; SUL= sulfonamides; TET= tetracyclines; PHE= phenicols; POL= polymyxins

Supplementary Table S8: MDR pattern in Gram-positive strains.

N. Classes	Antimicrobial Classes	Bacterial Genera and n. strains ()	Total strains
9	AMI, CEP, CAR, FLU, GLY, LIN, MAC, PEN, TET	*Enterococcus* (1)	1
8	AMI, CEP, CAR, GLY, LIN, MAC, PEN, TET	*Enterococcus* (1); *Staphylococcus* (1)	2
	AMI, CEP, CAR, FLU, GLY, LIN, MAC, PEN	*Enterococcus* (4)	4
	AMI, CEP, CAR, FLU, LIN, MAC, PEN, TET	*Staphylococcus* (1)	1
7	AMI, CEP, CAR, GLY, LIN, MAC, PEN	*Bacillus* (1)	1
	CEP, CAR, GLY, LIN, MAC, PEN, TET	*Bacillus* (1)	1
	AMI, CEP, GLY, LIN, MAC, PEN, TET	*Bacillus* (1)	1
	AMI, CEP, FLU, LIN, MAC, PEN, TET	*Enterococcus* (1)	1
	AMI, CEP, CAR, FLU, LIN, MAC, PEN	*Enterococcus* (1)	1
	AMI, CEP, CAR, FLU, GLY, LIN, PEN	*Staphylococcus* (1)	1
6	AMI, CEP, CAR, GLY, MAC, PEN	*Bacillus* (1)	1
	AMI, CEP, CAR, LIN, MAC, PEN	*Bacillus* (1); Enterococcus (7)	8
	AMI, CEP, GLY, LIN, MAC, PEN	*Staphylococcus* (1)	1
	CEP, CAR, GLY, LIN, MAC, PEN	*Staphylococcus* (1)	1
5	CEP, GLY, LIN, MAC, PEN	*Bacillus* (1); *Staphylococcus* (1)	2
	AMI, CEP, GLY, MAC, PEN	*Bacillus* (1); *Staphylococcus* (2)	3
	AMI, CEP, LIN, MAC, PEN	*Bacillus* (1); *Staphylococcus* (1)	2
	AMI, CEP, CAR, MAC, PEN	*Enterococcus* (2)	2
	AMI, CEP, CAR, LIN, PEN	*Enterococcus* (3); *Staphylococcus* (4)	7
	CEP, CAR, GLY, LIN, PEN	*Staphylococcus* (1)	1
	AMI, CEP, CAR, GLY, PEN	*Staphylococcus* (1)	1

	CEP, GLY, LIN, PEN, TET	*Staphylococcus* (1)	1
	AMI, CEP, GLY, LIN, PEN	*Staphylococcus* (1)	1
	AMI, CEP, FLU, GLY, MAC	*Staphylococcus* (1)	1
4	CEP, LIN, MAC, PEN	*Bacillus* (2)	2
	CEP, GLY, MAC, PEN	*Bacillus* (1)	1
	GLY, LIN, MAC, PEN	*Bacillus* (1); *Enterococcus* (1); *Staphylococcus* (1)	3
	AMI, CEP, LIN, PEN	*Bacillus* (4); *Staphylococcus* (4)	8
	AMI, CEP, MAC, PEN	*Bacillus* (1)	1
	AMI, CEP, CAR, PEN	*Enterococcus* (2); *Staphylococcus* (2)	4
	CEP, LIN MAC, TET	*Staphylococcus* (1)	1
	CEP, FLU, PEN, TET	*Staphylococcus* (2)	2
	CEP, GLY, LIN, PEN	*Staphylococcus* (1)	1
	AMI, CEP, CAR, PEN	*Staphylococcus* (1)	1
	AMI, CEP, GLY, PEN	*Staphylococcus* (2)	2
3	AMI, CEP, PEN	*Aerococcus* (2); *Bacillus* (5); *Enterococcus* (2); *Staphylococcus* (5)	14
	CEP, GLY, PEN	*Bacillus* (1); *Staphylococcus* (3)	4
	CEP, LIN, PEN	*Bacillus* (2); *Staphylococcus* (2)	4
	CEP, MAC, PEN	*Bacillus* (1); NI (1);	2
	LIN, MAC, PEN	*Staphylococcus* (1)	1
	CEP, GLY, MAC	*Staphylococcus* (1)	1
